# Mesenchymal Stromal Cell Secretome Up-Regulates 47 kDa CXCR4 Expression, and Induce Invasiveness in Neuroblastoma Cell Lines

**DOI:** 10.1371/journal.pone.0120069

**Published:** 2015-03-16

**Authors:** Vipin Shankar, Hiroki Hori, Kentaro Kihira, Qi Lei, Hidemi Toyoda, Shotaro Iwamoto, Yoshihiro Komada

**Affiliations:** Department of Pediatrics, Mie University Graduate School of Medicine, 2-174, Edobashi, Tsu, Mie, Japan; University of Florida, UNITED STATES

## Abstract

Neuroblastoma accounts for 15% of childhood cancer deaths and presents with metastatic disease of the bone and the bone marrow at diagnosis in 70% of the cases. Previous studies have shown that the Mesenchymal Stromal Cell (MSC) secretome, triggers metastases in several cancer types such as breast and prostate cancer, but the specific role of the MSC factors in neuroblastoma metastasis is unclear. To better understand the effect of MSC secretome on chemokine receptors in neuroblastoma, and its role in metastasis, we studied a panel of 20 neuroblastoma cell lines, and compared their invasive potential towards MSC-conditioned-RPMI (mRPMI) and their cytokine receptor expression profiles. Western blot analysis revealed the expression of multiple CXCR4 isoforms in neuroblastoma cells. Among the five major isoforms, the expression of the 47 kDa isoform showed significant correlation with high invasiveness. Pretreatment with mRPMI up-regulated the expression of the 47 kDa CXCR4 isoform and also increased MMP-9 secretion, expression of integrin α3 and integrin β1, and the invasive potential of the cell; while blocking CXCR4 either with AMD 3100, a CXCR4 antagonist, or with an anti-47 kDa CXCR4 neutralizing antibody decreased the secretion of MMP-9, the expression of integrin α3 and integrin β1, and the invasive potential of the cell. Pretreatment with mRPMI also protected the 47 kDa CXCR4 isoform from ubiquitination and subsequent degradation. Our data suggest a modulatory role of the MSC secretome on the expression of the 47 kDa CXCR4 isoform and invasion potential of the neuroblastoma cells to the bone marrow.

## Introduction

Neuroblastoma, a biologically heterogeneous tumor originating from the sympathetic nervous system, is the most common extra-cranial solid tumor in childhood and the most frequently diagnosed neoplasm during infancy [1, 2, 3]. About half of all patients presenting with neuroblastoma have disease dissemination at the time of diagnosis. The most common metastatic sites include the bone, bone marrow, liver and non-contiguous lymph nodes [1, 4]. Treatment of patients with disseminated neuroblastoma is one of the greatest challenges for pediatric oncologists, as the 5 year survival rate remains as low as 40–45%, despite advanced treatment options [5]. Disseminated disease often leads to fatal outcomes, and children with bone metastasis have a <7% survival rate [6, 7]. Forty to 50% of patients present with relapse even with complete remission after multi modal treatment including surgery, chemotherapy and radiation therapy [8]. Bone marrow is a major metastatic site in stage IV neuroblastoma, and is expected to precede bone metastasis. Evaluation of minimal residual disease in the bone marrow has been suggested as a predictor of treatment outcomes. [9, 10, 11].

A close interaction between metastatic tumor cells and the bone marrow micro environment has been proposed as a key step in the establishment of bone marrow metastasis in several tumor types such as breast and prostate cancer [12, 13, 14]. Mesenchymal stromal cells (MSCs), a group of multipotent cells in the bone marrow with self-renewal ability, has long been thought to play important roles in the progression and establishment of metastatic lesions in the bone marrow cavity in various tumors [15, 16, 17,18]. It is generally believed that MSCs exert their effects on cancer cells through secreted trophic factors, which provide a supportive microenvironment for cell survival, cell renewal, angiogenesis and migration [19]. Stromal cell derived factor 1 (SDF 1), or CXCL12 is an important member of the chemokine family, and a potent chemoattractant for hematopoietic stem cells and many leukocytes. CXCL12 represents a component of the bone marrow microenvironment secretome that is chiefly secreted in the bone marrow by the MSCs [20]. In addition to its physiologic functions of regulating hematopoietic progenitors homing to the bone marrow, and their retention within the bone marrow microenvironment, CXCL12 is involved in the proliferation, survival and the metastases of many different cancers [21, 22]. A wide distribution of CXCR4, the major receptor of CXCL12, on various types of tumors may account for neoplastic progression [23, 24, 25].

Previous studies using cell lines and primary cancer samples have shown correlations between high CXCR4 expression levels on neuroblastoma cells and increased occurrence of bone marrow metastases [26, 27]. Other studies have also shown that CXCR4 supports establishment of neuroblastoma primary tumors [28, 29]. However, there are a few studies that showed contradictory results [30, 31]. Therefore, additional investigations would be necessary to better understand the role of CXCR4—CXCL12 axis in neuroblastoma biology. The aim of this study is to understand the effect of MSC-secretome on the expression of CXCR4 and the metastatic potential of neuroblastoma cell lines.

In this study, we have investigated the expression of CXCR4 on 20 different neuroblastoma cell lines, and classified them on the basis of their invasive potential and CXCR4 expression profile. The results revealed a good correlation between the invasive potential and the expression of the 47 kDa CXCR4 isoform. We found an isoform-specific-over-expression of CXCR4, in neuroblastoma cell lines, upon exposure to the MSC secretome, and a protective role of the MSC secretome in the expression of the 47 kDa CXCR4 isoform. This regulatory role of the MSC secretome on the expression of the invasion-specific 47 kDa CXCR4 isoform could be a molecular target in the treatment of advanced neuroblastoma.

## Materials and Methods

### Neuroblastoma Cell Lines

20 Neuroblastoma cell lines, described previously, were used in the study. Table 1 shows the cell lines and the clinical features of patients from whom the cell lines were derived [32, 33]. The cells were cultured in RPMI-1640 (Sigma), supplemented with 10% FBS (GIBCO). The cells were maintained in a humidified atmosphere at 37°C with 5% CO_2_.

**Table 1:** Neuroblastoma cell lines and their clinical features.

No	Cell Line	Clinical Features					
		Patient DetailsAge (yr.mo)/Sex	Primary Site	Metastatic Site	Cell Line Origin	Stage	N-myc Amplification
1	GOTO	1.1/?	Adrenal Gland	uk	Adrenal Gland	uk	+
2	IMR 32	1.1/M	Abdomen	Brain	Brain	uk	+
3	INDEN	4.10/F	Adrenal Gland	Bone	Adrenal Gland	4	-
4	KP-N-RT	?	Uk	BM	BM	uk	+
5	KP-N-SI	5/M	Adrenal Gland	LN, Bone	Bone	4	-
6	LAN-1	2/M	Uk	BM	BM	4	+
7	LAN-5	0.4/M	Uk	BM	BM	uk	+
8	NB 19	1/F	Uk	BM	BM	4	+
9	NB Mass	0.7/M	Thorax	-	-	1	-
10	MNB-OZ	1.10/F	Abdomen	BM, LN, Bone	BM	4	+
11	SCMC-N4	?	Uk	uk	Brain	uk	+
12	SH-N-DZ	2/F	Uk	BM	BM	uk	uk
13	SHEP	?	Uk	uk	Brain	uk	uk
14	SJ-N-KP	5/?	Uk	BM	BM	uk	+
15	SJ-N-KS	Uk	uk	uk	uk	uk	uk
16	SJ-N-SD	Uk	Uk	uk	uk	uk	uk
17	SK-N-SH	4/F	Thorax	BM	BM	4	-
18	SMS-KAN	3/f	Pelvis	BM, LN	Pelvis	4	+
19	MNB-SU	1.6/F	Adrenal Gland	BM, LN, Bone	BM	4	uk
20	TGW	?	Adrenal Gland	uk	Adrenal Gland	uk	uk

*BM*: *Bone Marrow*, *LN*: *Lymph Node*, *uk*: *Unknown*

### Mesenchymal Stromal Cells (MSC)

Immortalized human telomerase reverse transcriptase transduced MSC cell line (MSC-TERT) [34] was cultured in RPMI-1640 (Sigma) supplemented with 10% FBS (GIBCO) and 0.01M hydrocortisone (Sigma). The cells were maintained in a humidified atmosphere at 37°C with 5% CO_2_.

### MSC conditioned RPMI (mRPMI)

MSC-TERT cells were cultured to 80% confluence. The cells were then washed with cold Phosphate Buffered Saline (PBS) (Sigma), and fresh RPMI-1640 was added. The media was harvested after 24 hours, centrifuged, and the supernatant was collected and stored at 4°C for further use.

### Transwell Invasion studies

Neuroblastoma cells, serum-starved for 24 hours were plated at a seeding density of 0.5 X 10^5^ cells per well onto 0.8 micron transwell meshes, coated with Basement Membrane Extract (BME) coat (Trevigen, Cat no #3455–096–03) at 0.5x to 1x dilution in 1x coating buffer (Trevigen, Cat no #3455–096–03). The reservoir chambers were set up with 150 μl of RPMI or mRPMI. The plates were incubated at 37°C in a 5% CO_2_ incubator for 12 hours. The cells invading across the mesh were harvested using 100 μl of 1x Cell Dissociation Solution (CDS) (Trevigen, Cat no #3455–096–05) containing 1.2 μl of Calcein AM (Molecular Probes, Cat no #C3100MP) solution (1.67 μg/μl in DMSO). The cell number was determined by comparing the fluorescence read in a fluorescence top reader (Perkin Elmer, Arvo 2) at 485 nm for excitation and 520 nm emission to a standard curve for each cell line. The normalized values were then used to calculate the invasion index.

For inhibition studies, either AMD 3100 (Sigma, Cat No# A5602) or anti-CXCR4 neutralizing antibody (R&D systems, Cat No# MAB170) was added to the medium during the serum starvation step. To identify the stimulatory effect of factors from the MSC, the serum starvation step was reduced to 12 hours followed by incubation in mRPMI for 12 hours.

### Flow-cytometric analysis

Neuroblastoma cells were cultured and harvested using Trypsin (Gibco, Cat No# 25200) and washed twice with cold PBA (PBS with 2% BSA). The cell number was adjusted to 1 X 10^7^ cells/ml in PBA. 50 μl of cells were incubated with 20 μl of the PE-conjugated anti-human CXCR4 antibody (R&D systems Cat No# FAB170P) or PE-conjugated anti-human CXCR5 antibody (R&D systems, Cat No# FAB190P) or PE-conjugated anti-human CXCR6 antibody (R&D systems, Cat No# FAB699P) at room temperature for 30 minutes in dark. Control PE-conjugated mouse IgG_2a_ (Acris antibodies, Cat No# SM11R) or Control PE-conjugated mouse IgG_2b_ (Acris antibodies, Cat No# SM12R) was used for negative controls. After incubation with the antibody, the cells were washed three times with PBA and analyzed on a BD FACSCaliber flow cytometer using CellQuest software (BD Biosciences). One thousand events were counted for each sample, and the mean fluorescent intensity from three experiments was calculated.

### Protein Immune-precipitation

CXCR4 was immune-precipitated using the Pierce Crosslink Immunoprecipitation kit (Thermo Scientific, Cat No# 26147), according to manufacturer’s instructions. 10 μg/ml anti-CXCR4 antibody (Thermo Scientific, Cat No #PA1–12543) was bound to 20 μl of Pierce Protein A/G Plus Agarose resin in a spin column, and cross linked with 2.5 mM Disuccinimidyl suberate (DSS) crosslinker. Cell lysates were pre-cleared using control agarose resin, and added to the antibody bound agarose columns. After overnight incubation at 4°C, the antigen was eluted using the elution buffer.

### Cell surface protein extraction

Cell surface proteins were extracted using the Pierce Cell surface protein isolation kit (Thermo Scientific, Cat No# 89881), according to manufacturer’s instructions. Briefly, 90% confluent cells in 75 cm^2^ cell culture flasks, were biotinylated and then lysed using the kit-provided lysis buffer. The labeled proteins were then isolated and eluted using a NeuterAvidin Agarose column.

### Western blot studies

Western blot studies were carried out, as previously described [35, 36]. Cells were harvested and 1 X 10^7^ cells were washed twice in PBS and solubilized in 1 ml lysis buffer (50 mN Tris base, 150 mM NaCl, 1% Triton X-100 pH 7.5). The lysates were centrifuged at 14,000 rpm at 4°C for 30 minutes. The supernatants were separated and used for western blot analysis. Equal amounts of proteins, as determined by BCA protein assay (Thermo Scientific Cat No #23225), from the total cell extract, cell surface protein extract or from immune precipitated samples were loaded in wells in a 12.5% polyacrylamide gel followed by electrophoresis at 120 constant volts for 90 minutes. Kaleidoscope Precision Plus protein standards (BioRad Cat no # 161–0375) were run alongside for size determination. The resolved proteins were transferred to PVDF membranes (life technologies) using iBlot (life technologies), following the manufacturer’s instructions. Membranes were blocked either in 5% non-fat milk in TBST containing 0.1% Tween-20 or SuperBlock T20 (TBS) Blocking Buffer (Thermo Scientific, Cat No# 37536) for 30 minutes, followed by a wash in TBST. The membranes were then incubated with primary antibody (1:1000–3000 dilution) overnight at 4°C. (The antibodies used are shown in Table 2.) After washing in TBST, the membranes were incubated with HRP conjugated secondary antibody (1:1000–3000 dilution) for 30 minutes at room temperature. The membranes were then washed, and incubated with a chemiluminescent substrate (Western lightening chemiluminescence reagent plus, Perkin Elmer Life Science) for one minute at room temperature. The bands were visualized on LAS 3000 mini (Fujifilm, Tokyo, Japan). The blots were analyzed and quantified using imageJ (Maryland, USA).

**Table 2:** Antibodies used in western blot studies.

No	Antibody	Species Reactivity	Clone	Host	Maker	Catalog No
1	Anti-CXCL12 antibody	Human	Polyclonal	Rabbit	Sigma-Aldrich	AV07031
2	Anti-CXCR4 antibody	Human	Polyclonal	Rabbit	Abcam	Ab2090
3	Anti-CXCR4 antibody	Human	Polyclonal	Rabbit	Pierce	PA1–12543
4	Anti CXCR4 antibody	Human	Monoclonal	Mouse	Abnova	H00007852-M05
5	Anti-CXCR5 antibody	Human	Polyclonal	Rabbit	BioVision	3419–100
6	Anti-CXCR6 antibody	Human	Polyclonal	Rabbit	Enzo	ADI-905–276
7	Integrin Alpha-3 antibody	Human	Polyclonal	Rabbit	ABBIOTEC	251177
8	Integrin Beta-1 antibody	Human	Polyclonal	Rabbit	LSBio	LS-C137443
9	Ubiquitin/Ubiquitin +1 antibody	Human	Monoclonal	Mouse	R&D Systems	MAB 701
10	Anti-actin antibody	Human	Polyclonal	Rabbit	Sigma-Aldrich	A2066
11	ECL Anti-mouse IgG HRP Linked	Mouse	Polyclonal	Sheep	GE	NA931V
12	ECL Anti-rabbit igG HRP linked	Rabbit	Polyclonal	Donkey	GE	NA934V

### Far western blot studies

Immune precipitated CXCR4 from the cell lines were electrophoresed on 12.5% polyacrylamide gels, under non-reducing conditions, and transferred on to PVDF membrane, as described before. After blocking with blocking buffer for 30 minutes at room temperature, the membranes were incubated overnight at 4°C with human CXCL12 (Miltenyi Biotec, Cat No #130–093–997) in TBS-T. The membranes were then washed, blocked again and incubated with anti-CXCL12 antibody (Sigma Aldrich, Cat No# AV07031), overnight at 4°C. The membranes were washed in TBS-T and then incubated with 1/2000 dilution of HRP conjugated secondary antibody for 30 minutes at room temperature. The bands were visualized and analyzed as described before.

### Gelatin zymography

Pro-MMP-9 was assayed using the gelatin zymography kit (Cosmo Bio, Cat no# AK-45) according to manufacturer’s instructions. Briefly, the culture supernatants from the cell culture were harvested, centrifuged and filtered to remove any cell debris. Equal amounts of total protein was added to gelatin impregnated poly-accrylamide gel and electrophoresis carried out under non-reducing conditions. The gels were then incubated in the kit-provided reaction buffer for 40 hours at 37°C. After incubation, the gels were stained in a staining solution for 30 minutes at room temperature, and then de-stained with de-staining solution (acetic acid:methanol:water = 5:30:65) for 20 minutes to an hour to get clear bands. The gels were scanned under Fuji-xerox (Apeos Port V C6675) at 300 dpi and the images were analyzed using imageJ.

### Statistical analysis

Data represent the mean ± standard error of the indicated number of independent experiments. One sample student’s t-test was used to determine whether samples were representative of a given experimental condition. One way ANOVA with Bonferroni post tests was used to compare between groups. A p value less than 0.05 was considered as statistically significant.

## Results

### Neuroblastoma cell lines are heterogeneous in their invasive potential

**Fig 1:**
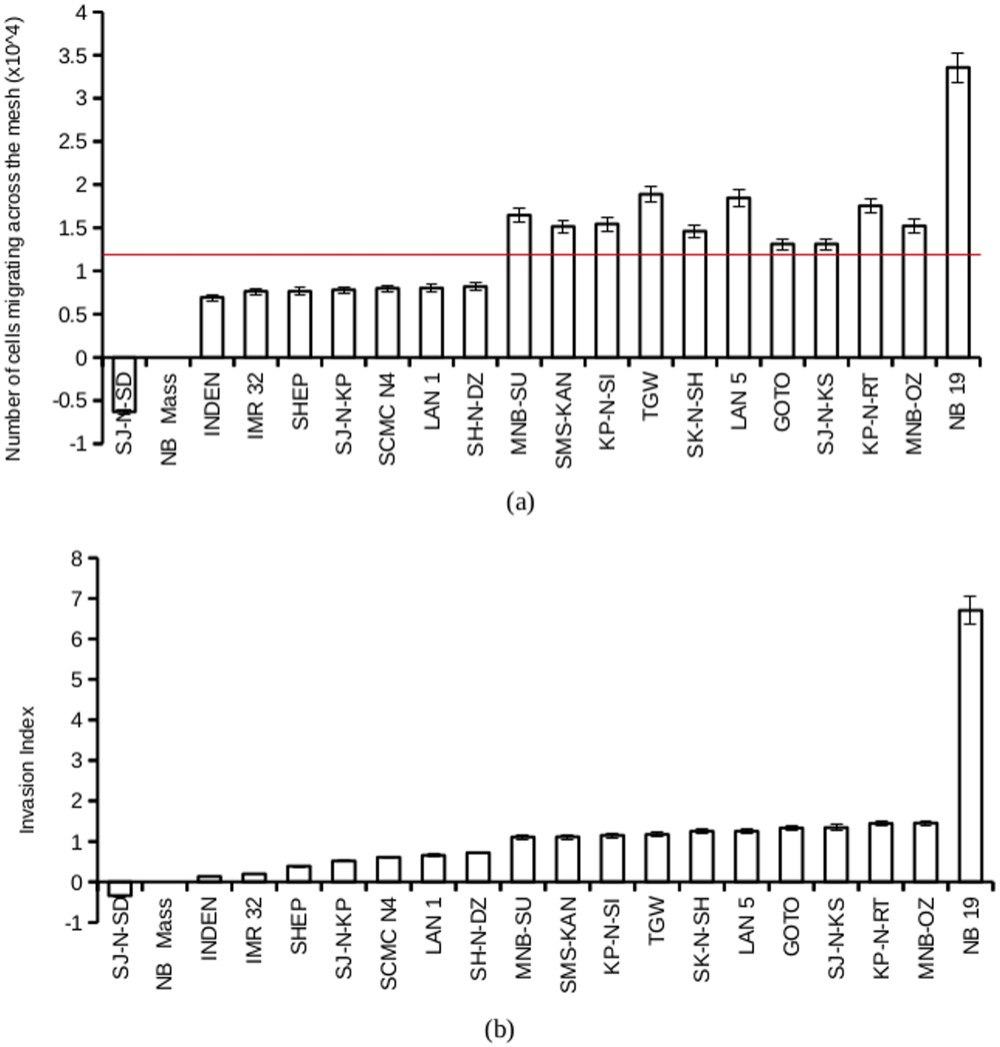
Invasive potential of neuroblastoma cell lines. a. 20 neuroblastoma cell lines were serum-starved for 24 hours, and plated at a seeding density of 0.5 X 10^5^ cells per well on to 0.8 micron cell culture inserts, coated with Basement Membrane Extract (BME) coat at 0.5x to 1x dilution. The number of cells crossing the mesh towards RPMI (control) or mRPMI were counted fluorimetrically using calcein AM after 12 hours. Plotted is the average number of cells, for each cell line from 7 experiments, migrating across the mesh. The red line indicates the mean value. Cell lines higher than the mean value were classified as highly invasive and those below the mean value as less invasive cell lines. b. Invasion index was calculated as the ratio of number of cells invading towards mRPMI versus the number of cells invading towards RPMI. Plotted is the mean invasion index from 7 experiments in increasing order. Cell lines with an invasion index of 1 or higher was classified as highly invasive cell lines and those with an invasion index of less than 1 was classified as less invasive.

Neuroblastoma cells were assayed for their invasive potentials using a transwell invasion experiment setting. A cell line NB 19 showed most robust migration across the mesh, while cell lines NB mass and SJ-N-SD showed least robust migration across the mesh (Fig. 1a). The invasion index was calculated as the ratio of the number of cells invading towards mRPMI versus the number of cells invading towards RPMI. Neuroblastoma cell lines showed heterogeneity in their invasion indices (Fig. 1b). NB 19 showed a very high invasion index compared to other cell lines. NB mass, and SJ- N- SD showed very low invasion indices.

Cell lines were classified into two groups according to the number of cells migrating across the mesh. Cell lines which showed cell migration above the mean value were classified into the highly invasive group, and those cell lines that showed cell migration below the mean were classified into the less invasive group. Invasion index for each cell line was also used in the classification. Cell lines with an invasion index greater than 1 were classified as highly invasive and those with an invasion index less than 1 was classified as less invasive. Both classifications gave identical results. NB 19, MNB-OZ, KP-N-RT, SJ-N-KS, GOTO, LAN 5, SK-N-SH, TGW, KP-N-SI, SMS-KAN and MNB-SU were classified as highly invasive cell lines, and SH-N-DZ, LAN 1, SCMC N4, SJ-N-KP, SHEP, IMR 32, INDEN, NB mass and SJ-N-SD, were grouped as the less invasive cell lines.

### Neuroblastoma cell lines are heterogeneous in their CXCR4 expression

**Fig 2:**
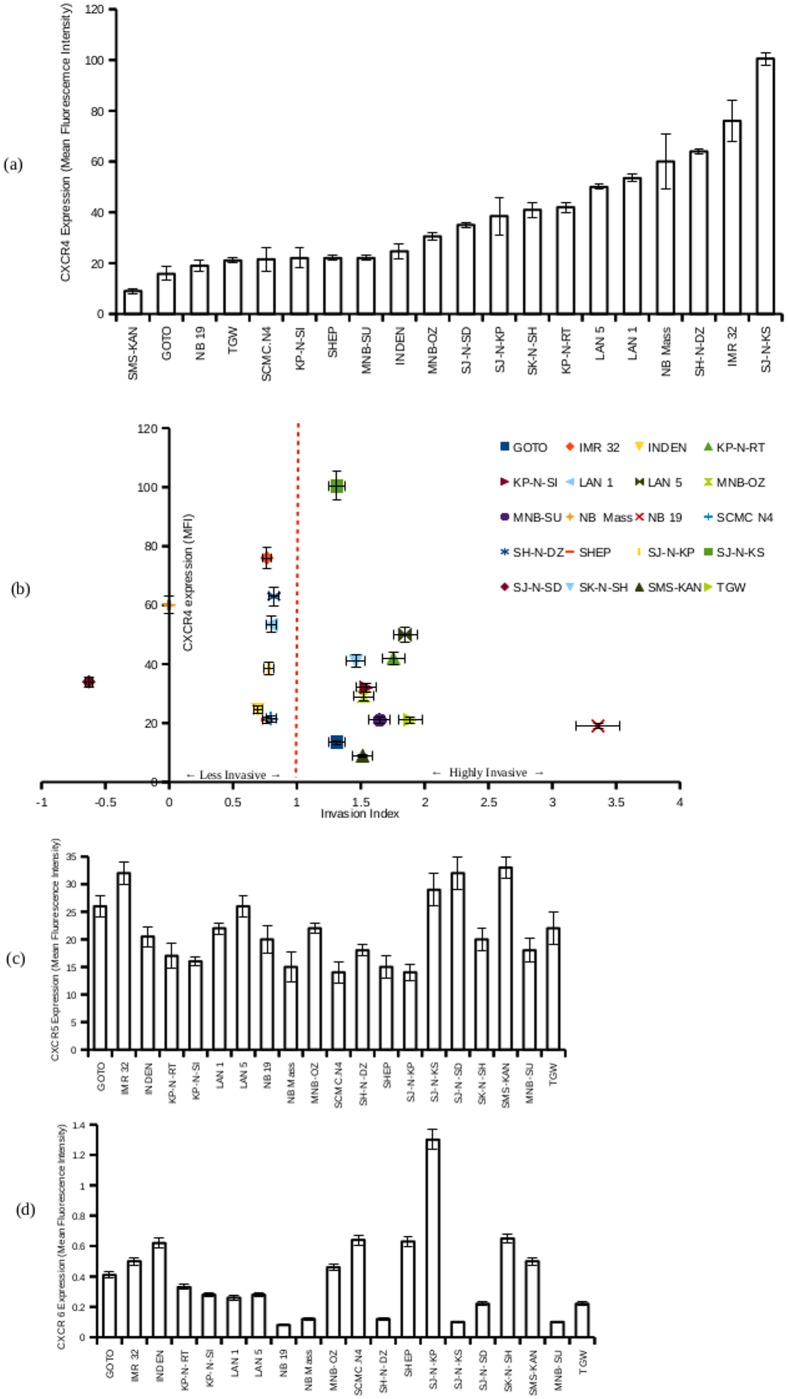
Expression of cytokine receptors CXCR4, CXCR5 and CXCR6 in neuroblastoma cell lines. a. Cell surface expression of CXCR4 on neuroblastoma cell lines was assayed by flow cytometry. Cells were harvested using trypsin, washed and incubated with PE-conjugated anti-CXCR4 antibody. After 30 minutes of incubation, the cells were washed and analyzed on a BD FACSCaliber flow cytometer using CellQuest. One thousand events were counted for each sample and the mean fluorescent intensity (MFI) from three experiments were calculated. Plotted is the MFI in increasing order. b. Plotted are the relative total CXCR4 expressions (MFI from 3 experiments) (y axis) and the invasion indices (x axis) of 20 neuroblastoma cell lines. c. Surface expression of CXCR5, plotted as MFI from three experiments. d. Surface expression of CXCR6, plotted as MFI from three experiments.

In order to study whether total CXCR4 expression had any role in determining the invasive potential of neuroblastoma cell lines, the total CXCR4 expression was assayed by flow-cytometry. Neuroblastoma cell lines showed heterogeneity in the expression levels of CXCR4 with SJ-N-KS showing the highest expression level and SMS-KAN showing the least (Fig. 2a). Correlation analysis did not derive any significant association (r = -0.253, r^2^ = 0.0641) between total CXCR4 expression and invasion index (Fig. 2b).

CXCR5 and CXCR6 expression levels were also measured using the same method. The expression levels of CXCR5 and CXCR6 showed little variance among the cell lines (Fig. 2c, Fig. 2d).

### CXCR4 structural heterogeneity in Neuroblastoma cell lines

**Fig 3:**
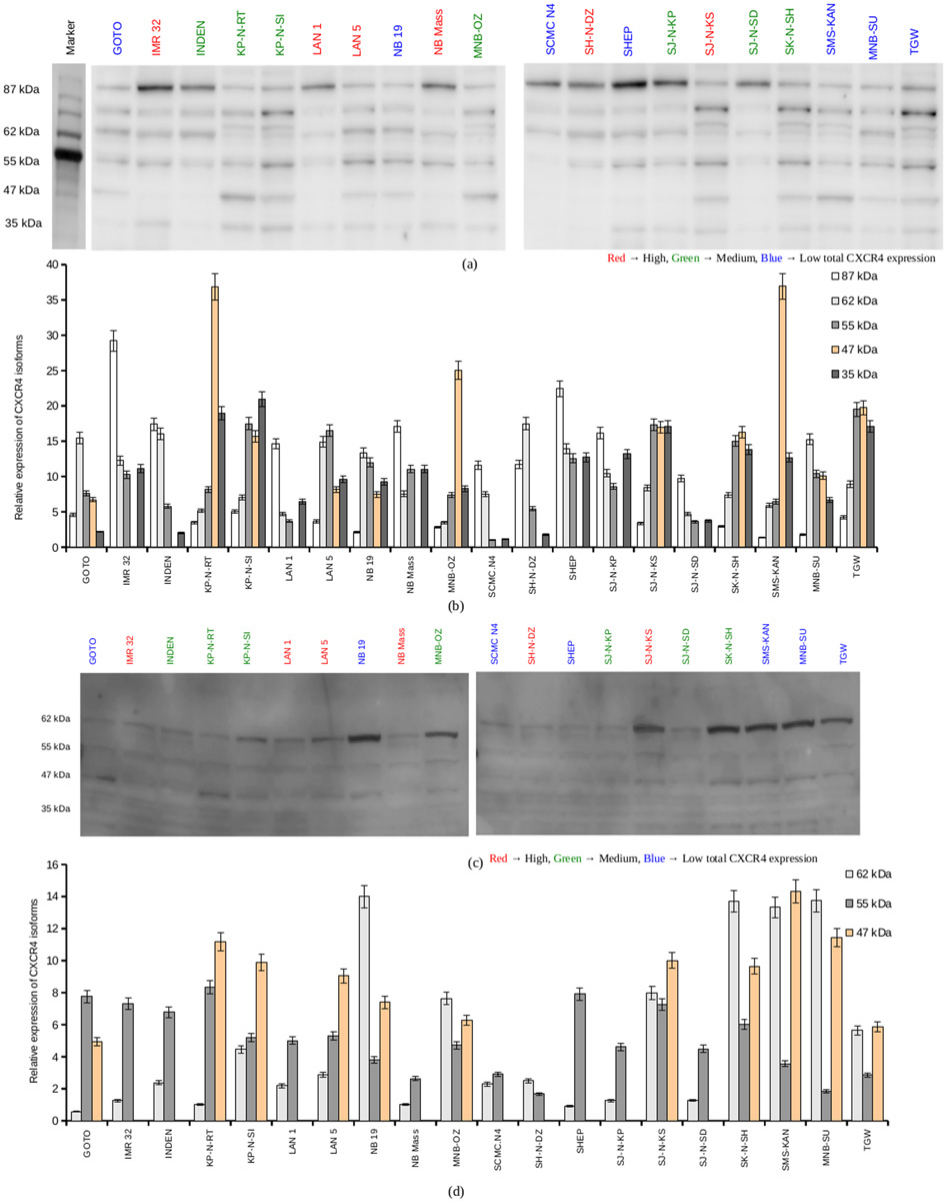
Structural heterogeneity of CXCR4 in neuroblastoma cell lines. a. Western blot detection of CXCR4 in 20 neuroblastoma cell lysates. 1 X 10^7^ cells were washed twice in PBS and solubilized in 1 ml of lysis buffer. The lysates were centrifuged at 14,000 rpm at 4°C for 30 minutes. Equal amounts of total proteins, as determined by BCA assay, were loaded on to a 12.5% polyacrylamide gel and electrophoresed. The proteins were then blotted on to a PVDF membrane and CXCR4 was detected using an anti-CXCR4 polyclonal antibody and a HRP conjugated secondary antibody. Shown in figure is a representative result from 5 experiments. b. The bands from western blot studies were quantified using imageJ. Plotted is the mean relative expression of CXCR4 isoforms in the whole cell lyaste from 5 experiments. c. CXCR4 was immune precipitated using the Pierce Crosslink Immunoprecipitation kit with anti-CXCR4 antibody. The precipitate was electrophoresed and blotted on to PVDF membrane. Protein detection was carried out using a second anti-CXCR4 antibody. Shown in figure is a representative result from 5 experiments. d. The bands from western blot studies were quantified using imageJ. Plotted is the mean relative expression of CXCR4 isoforms in the CXCR4 immune precipitate from 5 experiments.

In order to assess whether any specific isoform of CXCR4 might be responsible for the highly invasive phenotype in neuroblastoma, western blot detection of CXCR4 isoforms using a polyclonal anti-CXCR4 antibody was carried out. Multiple isoforms were detected in whole cell lysates (Fig. 3a), with most cells expressing the 87 kDa, 62 kDa, 55 kDa and the 35 kDa isoforms. Highest heterogeneity was observed in the expression of the 47 kDa isoform with only 11 of the 20 cell lines expressing it (Fig. 3a, Fig. 3b) (Table 3).

**Table 3:** Invasion potential and CXCR4 isoforms expressed in neuroblastoma cell lines.

No	Cell Line	Group	CXCR4 isoform expressed				
			87 kDa	62 kDa	55 kDa	47 kDa	35 kDa
1	GOTO	Highly invasive	✓	✓	✓	✓	✓
2	IMR 32	Less invasive	✓	✓	✓		✓
3	INDEN	Less invasive	✓	✓	✓		
4	KP-N-RT	Highly invasive	✓		✓	✓	✓
5	KP-N-SI	Highly invasive		✓	✓	✓	✓
6	LAN-1	Less invasive	✓	✓	✓		
7	LAN-5	Highly invasive	✓	✓	✓	✓	✓
8	NB 19	Highly invasive	✓	✓	✓	✓	✓
9	NB Mass	Less invasive	✓	✓	✓		
10	MNB-OZ	Highly invasive	✓		✓	✓	✓
11	SCMC-N4	Less invasive	✓	✓			
12	SH-N-DZ	Less invasive	✓	✓			
13	SHEP	Less invasive	✓	✓	✓		
14	SJ-N-KP	Less invasive	✓	✓	✓		
15	SJ-N-KS	Highly invasive	✓		✓	✓	✓
16	SJ-N-SD	Less invasive	✓				
17	SK-N-SH	Highly invasive	✓	✓	✓	✓	✓
18	SMS-KAN	Highly invasive	✓		✓	✓	✓
19	MNB-SU	Highly invasive	✓	✓	✓	✓	✓
20	TGW	Highly invasive	✓		✓	✓	✓

To confirm the identity of the expressed bands as CXCR4, CXCR4 immunoprecipitated from the whole cell lysates were subjected to western blot detection, with a second anti-CXCR4 polyclonal antibody. 35 kDa, 55 kDa and the 62 kDa isoforms were found to be expressed in both the less invasive cell lines and the highly invasive cell lines, whereas the 47 kDa was expressed only in the highly invasive cell lines NB 19, MNB-OZ, SK-N-SH, GOTO, KP-N-RT, KP-N-SI, LAN 5, SJ-N-KS, SMS-KAN, MNB-SU and TGW (Fig. 3c, Fig. 3d). In contrast, the 47 kDa isoform was not expressed in any of the less invasive cell lines (Fig. 3a, Fig. 3c).

### The 47 kDa CXCR4 isoform may play an important role in bone marrow metastasis of neuroblastoma

**Fig 4:**
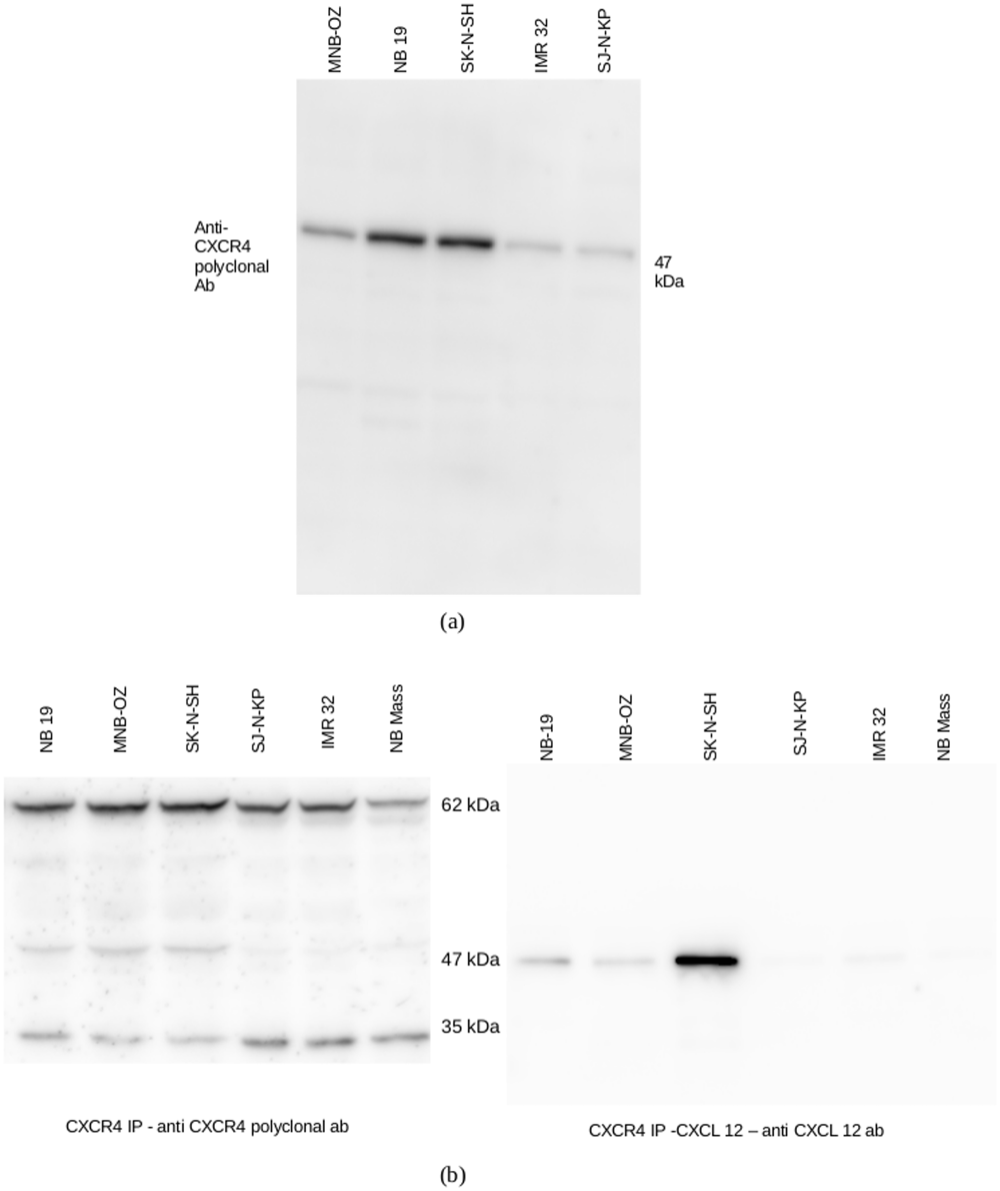
Localization and CXCL12 reactivity of CXCR4. a. The membrane proteins from 3 highly invasive neuroblastoma cell lines, MNB-OZ, NB 19 and SK-N-SH, and 2 less invasive cell lines, IMR 32 and SJ-N-KP were extracted, electrophoresed and blotted on to PVDF membrane. Protein detection was carried out using an anti-CXCR4 polyclonal antibody. Shown in figure is the representative result from 5 experiments. b. CXCR4 immune-precipiate from 3 highly invasive (NB 19, MNB-OZ & SK-N-SH) and 3 less invasive (SJ-N-KP, IMR 32 & NB Mass) cell lines were electrophoresed and blotted on to PVDF membrane. The membrane was incubated overnight with human CXCL12 in TBS-T at 4°C. The blot was washed and protein detection was carried out with anti-CXCL12 antibody. Shown in figure is the representative result from 3 experiments.

To determine the expression of CXCR4 on the cell membrane, the cell membrane was extracted from cell lines MNB-OZ, NB 19, SK-N-SH, IMR 32 and SJ-N-KP. The membrane proteins were then subjected to western blot analysis. Only the 47 kDa fraction was found to be expressed in the membrane fraction (Fig. 4a).

To ascertain any functional differences between the different isoforms of CXCR4 in neuroblastoma cell lines, CXCR4 immunoprecipitates from 3 highly invasive cell lines NB 19, MNB-OZ and SK-N-SH and 3 less invasive cell lines, NB Mass, SJ-N-KP and IMR 32, were subjected to far western blot analysis with CXCL12. CXCL12-CXCR4 complex, was observed only with the 47 kDa CXCR4 isoform in the 3 cell lines from the highly invasive group, while none of the cell lines from the less invasive group showed any significant CXCL12-CXCR4 complex formation (Fig. 4b).

**Fig 5:**
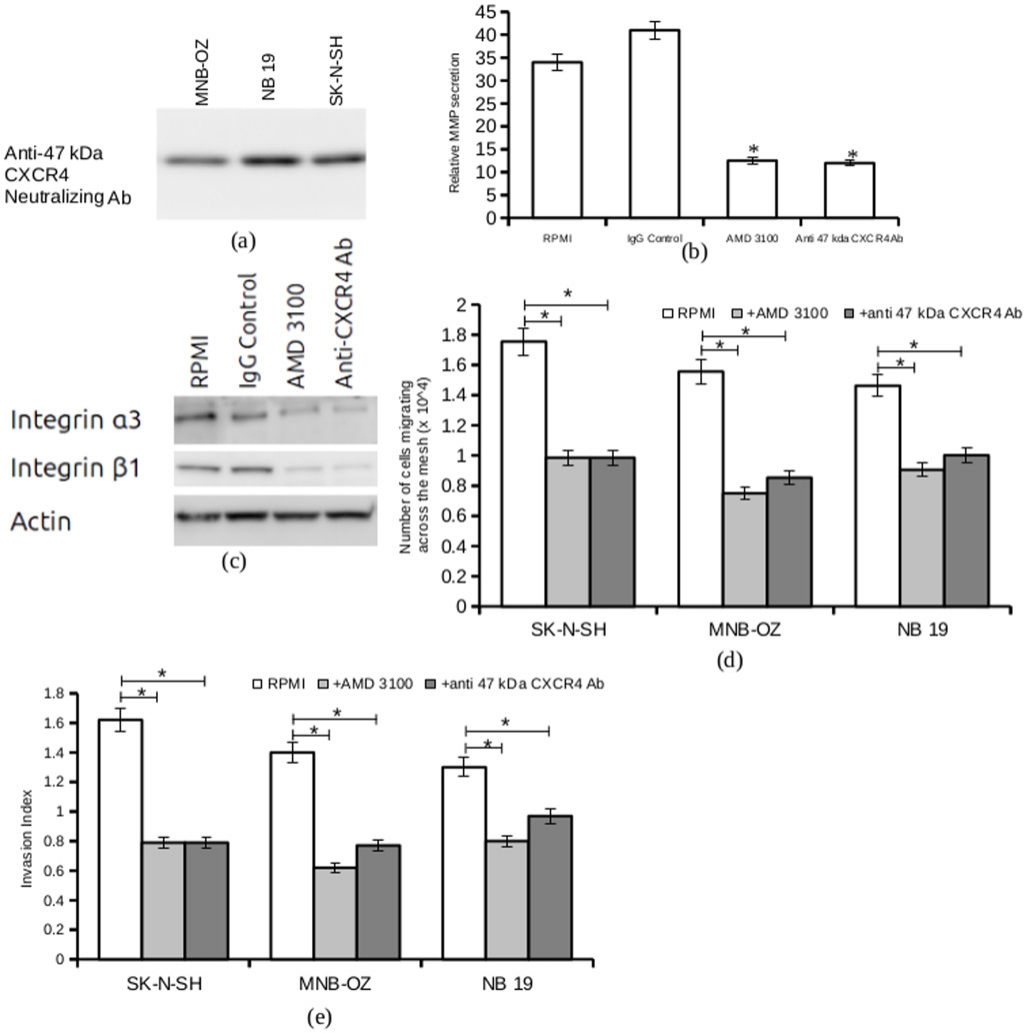
CXCR4 blocking assays. a. Total cell lysate from MNB-OZ, NB 19 and SK-N-SH cells were electrophoresed and blotted on to PVDF membrane, and protein detection was carried out with anti-47 kDa neutralizing antibody. Shown in figure is the representative result from 5 experiments. b. 80% confluent SK-N-SH cells were harvested and incubated in either RPMI, RPMI + IgG control, RPMI + AMD 3100 or RPMI + anti-47 kDa neutralizing antibody. The supernatants were harvested after 24 hours, centrifuged and subjected to gelatin zymography. The gels were analyzed using imageJ and the relative MMP-9 secretion was calculated from 3 experiments (* p < 0.05). c. Total cell lysates from SK-N-SH cells either serum-starved (control), treated with IgG control (negative control), RPMI + AMD 3100 or RPMI + anti-47 kDa neutralizing antibody was subjected to western blot analysis. Protein detection was carried out with anti-integrin α3 or anti-integrin β1 antibodies. Anti-actin antibody was used for loading control. Shown in figure is the representative result from 3 experiments. d. Transwell invasion studies were carried out with cell lines SK-N-SH, MNB-OZ and NB 19. Cells were serum-starved (control), treated with 10 μM AMD 3100 and treated with anti-47kDa CXCR4 neutralizing antibody. Plotted is the mean number of cells migrating across the mesh. (* p<0.05) e. Invasion index of cell lines SK-N-SH, MNB-OZ and NB 19, serum-starved (control) and treated with AMD 3100 and anti-47 kDa CXCR4 neutralizing antibody, were analyzed using transwell invasion assays. Plotted is the normalized invasion index values obtained from 5 experiments (* p < 0.05).

To further confirm the role of CXCR4 in the invasiveness of neuroblastoma cells towards mRPMI, all CXCR4 isoforms were blocked using 10 μM AMD 3100, a CXCR4 antagonist [37, 38], and the 47 kDa isoform was specifically blocked with an anti-47kDa CXCR4 neutralizing antibody. The specificity of the neutralizing antibody was confirmed by western blot analysis (Fig. 5a). Semi- confluent cells were harvested, washed, and incubated in RPMI containing either 10μM AMD 3100 or the anti-47 kDa CXCR4 neutralizing antibody. After incubation for 10 hours, the cells were harvested and the supernatant assayed for secreted Matrix Metalloproteinase 9 (MMP-9), a key enzyme in extracellular matrix remodeling and tumor progression.

A significant reduction in the levels of secreted MMP-9, compared to control levels, was observed in neuroblastoma cell lines treated either with AMD 3100 or with anti-47 kDa CXCR4 neutralizing antibody (Fig. 5b). Western blot analysis of whole cell lysates from the CXCR4 blocking experiment showed reduced expression of integrin α3 and integrin β1, both of which are deemed to be important [39, 40] in the initial stages of metastasis in neuroblastoma (Fig. 5c).

A transwell invasion experiment showed significant decreases in the invasion indices of highly invasive cell lines NB 19, SK-N-SH and MNB-OZ under CXCR4 block conditions (Fig. 5d, Fig. 5e). Invasion indicies of the less invasive neuroblastoma cell lines also showed a slight decrease, but the decrease was not as significant as that observed with the highly invasive cell lines.

### Pretreatment with MSC secretome increases the invasive potential in neuroblastoma cell lines

To investigate whether the bone marrow derived factors play a role in the organ specific metastasis of neuroblastoma cells to the bone marrow, neuroblastoma cells were pre-treated with mRPMI for 12 hours, and then assayed for CXCR4 expression levels, secreted MMP-9 levels, integrin expression levels and invasion indices.

**Fig 6:**
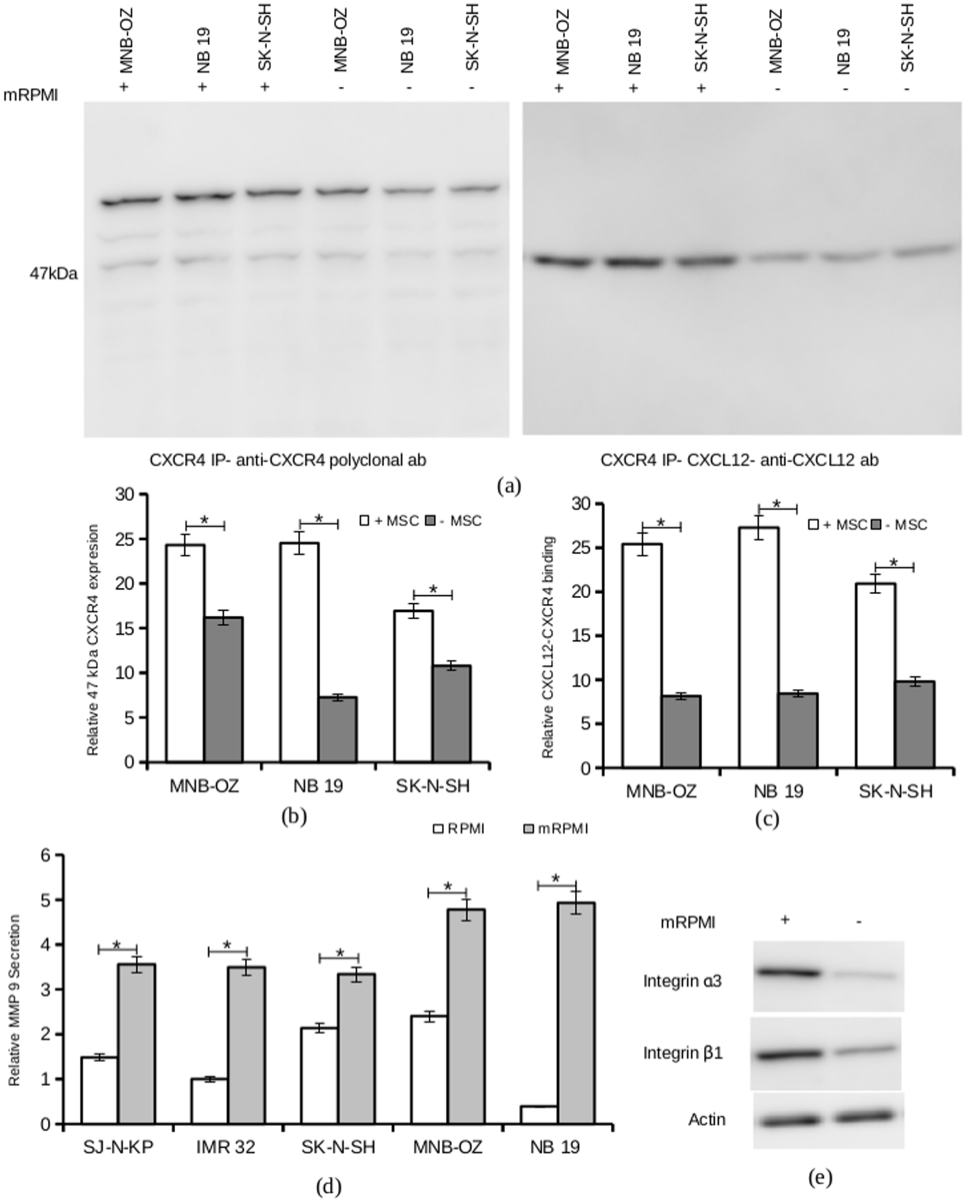
Effect of MSC secretome on neuroblastoma cell lines. a. (left panel) CXCR4 immune precipitates from cell lines MNB-OZ, NB 19 and SK-N-SH, serum-starved (control) and pre-treated with mRPMI were subjected to western blot analysis. Protein detection was carried out using anti-CXCR4 polyclonal antibody. Shown in figure is the representative result from 3 experiments. (right panel) CXCR4 immune precipitates from cell lines MNB-OZ, NB 19 and SK-N-SH, serum-starved (control) and pre-treated with mRPMI were electrophoresed and blotted on to PVDF membrane. The membrane was incubated with human CXCL12 overnight at 4°C. Protein detection was carried out using anti-CXCL12 antibody. Shown in figure is the representative result from 3 experiments. b. The blots were quantified using imageJ. Plotted are the mean relative expression of CXCR4 from 3 experiments. (* p < 0.05) c. Plotted are the mean relative CXCR4 bound CXCL12 from 3 experiments. (* p < 0.05). d. Culture supernatants from cells serum-starved (control) and pre-treated with mRPMI were harvested and subjected to gelatin zymography. The gels were scanned and the images were analyzed using imageJ. The relative secretion of MMP-9 was calculated from 3 experiments (*p < 0.05). e. Total cell lysate from serum-starved and mRPMI treated SK-N-SH cells were subjected to western blot analysis, and protein detection was carried out using anti-integrin α3 or anti-integrin β1 antibodies. Anti-actin antibody was used for loading control.

Immunoprecipitated CXCR4 from serum-starved or mRPMI pre-treated neuroblastoma cells were subjected to western blot analysis for the determination of CXCR4 isoforms. No significant change in the expression levels of the 62 kDa isoform was observed with mRPMI treatment. Interestingly, the expression of the 47 kDa isoform increased significantly with mRPMI pre-treatment, suggesting a direct involvement of the factors from MSC in neuroblastoma metastasis (Fig. 6a, Fig. 6b). Far western blot analysis showed increased CXCL12-CXCR4 binding with mRPMI (Fig. 6a, Fig. 6c).

Pre-treatment with mRPMI increased the levels of secreted MMP-9 compared to the control (serum-starved) neuroblastoma cells (Fig. 6d). This increase in MMP-9 secretion was observed in both the less and the highly invasive cell line groups. An increase in the expression of integrin α3 and integrin β1 was also observed with mRPMI pretreatment (Fig. 6e).

**Fig 7:**
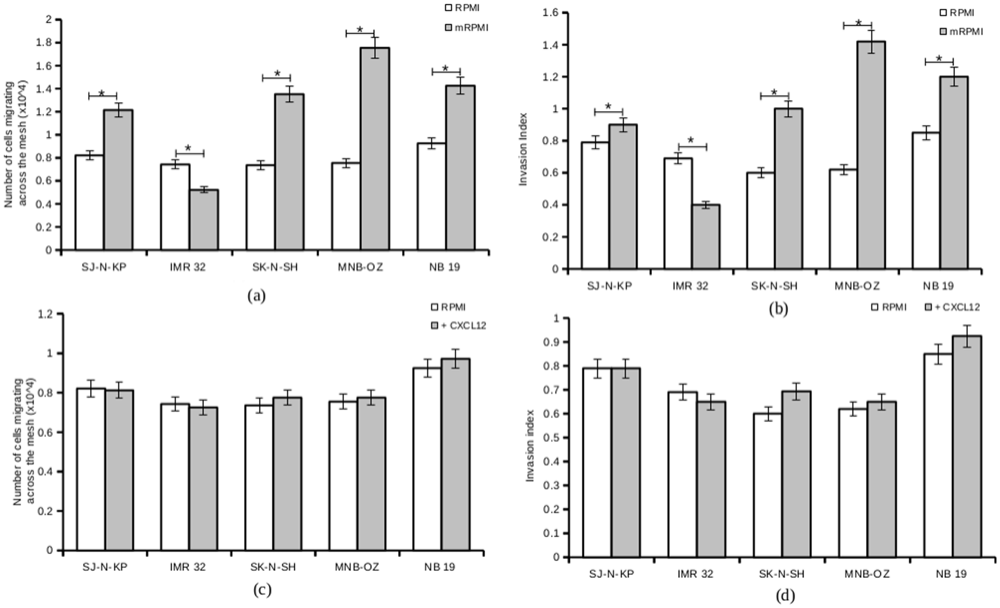
Effect of CXCL12 and mRPMI on invasion in neuroblastoma cells. a. Neuroblastoma cells serum-starved (control) and pre-treated with CXCL12, were assayed for invasive potential using transwell invasion assays. Plotted is the mean number of cells migrating across the mesh from 5 experiments. b. Invasion index of neuroblastoma cell lines serum-starved (control) and pretreated with CXCL12. Plotted is the invasion index calculated from 5 experiments. c. Neuroblastoma cells serum-starved (control) and pre-treated with mRPMI, were assayed for invasive potential using transwell invasion assays. Plotted is the mean number of cells migrating across the mesh from 5 experiments. (*p<0.05) d. Invasion index of neuroblastoma cell lines serum-starved (control) and pretreated with mRPMI. Plotted is the invasion index calculated from 5 experiments. (*p<0.05)

An increased number of cells migrating across the mesh and an increased invasion index were observed with cell lines pre-treated with mRPMI. A 10 hour incubation in mRPMI almost doubled the invasion index in MNB-OZ, which showed the most prominent increase (Fig. 7a, Fig. 7b). NB 19 and SK-N-SH pretreated with mRPMI also showed very high increases in their invasion indices. SJ-N-KP, a cell line from the less invasive group, showed a slight increase in the invasion index with mRPMI pre-treatment. Only one cell line, IMR 32 showed a decrease in the invasion index when pre-treated with mRPMI.

To analyze whether the increase in invasion potential of cell lines was induced solely by CXCL12, neuroblastoma cells were pretreated with CXCL12 and subjected to transwell invasion studies. The highly invasive cell lines NB 19, MNB-OZ and SK-N-SH showed a marginal increase in invasive potential, whereas the less invasive cell lines showed little or no increase (Fig. 7c, Fig. 7d).

### mRPMI treatment protected 47 kDa CXCR4 isoform from ubiquitination

To evaluate possible protective effects of MSC on CXCR4 function in neuroblastoma cells, CXCR4 immunoprecipitated from the SK-N-SH cell line, which were either serum-starved or pre-treated for 10 hrs with mRPMI, were subjected to western blot analysis for ubiquitin.

**Fig 8:**
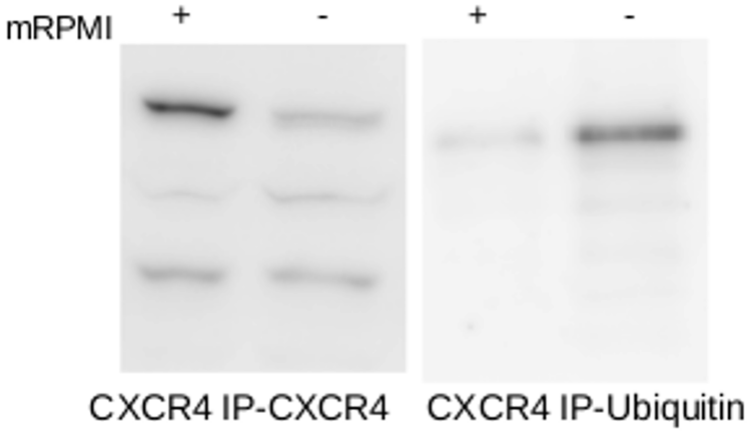
Effect of mRPMI on CXCR4 ubiquitination. CXCR4 immune precipitates from serum-starved (control) and mRPMI treated SK-N-SH cells were subjected to western blot analysis. Protein detection was carried out using anti-ubiquitin antibody. Shown is the representative result from 3 experiments.

Ubiquitination rates on CXCR4 were significantly lower in the presence of mRPMI, while cells not treated with mRPMI showed high ubiquitination of CXCR4 (Fig. 8).

## Discussion

Organ-specific metastasis may be the result of several sequential and selective steps which are coordinated and regulated by different tumor cell properties. To establish metastasis at preferential organs or tissues, tumor cells should manifest the ability to use stimuli to direct them into the preferred tissues, and favor their proliferation. Mutually exclusive factors like expression of chemotactic factors at the metastatic site, expression of adhesion molecules and the availability of growth and proliferation factors may support site-specific metastasis of various tumors. However little knowledge is available on how the metastatic host site modulates the tumor and triggers metastasis.

The present study addresses the possible regulatory role of chemotactic stimuli, in the migration of the neuroblastoma cells to the bone marrow and the establishment of bone marrow metastatic niche. This study aims to test the hypothesis that neuroblastoma metastasis uses a mechanism similar to the homing of hematopoietic stem cells to the bone marrow niche. A panel of 20 neuroblastoma cell lines was examined for their invasion potential towards MSC-conditioned RPMI and their CXCR4 expression profiles.

We found an isoform-specific over expression of CXCR4 in neuroblastoma cells triggered by the MSC secretome. The over expression of the 47 kDa CXCR4 isoform is suspected to mediate invasiveness through changes in integrin expression and MMP secretion. Earlier studies had demonstrated the role of CXCR4 in metastasis of different tumors, but the mechanism by which CXCR4 is up-regulated is not fully elucidated. In this study, we explored the MSC secretome—tumor interactions, and for the first time demonstrated a positive regulation of the 47 kDa CXCR4 isoform, in neuroblastoma, which might be a key step in the process of metastasis. This result gives new insights into neuroblastoma biology, especially in the process of bone marrow metastasis. Since the 47 kDa isoform of CXCR4 was found only in cell lines expressing the highly invasive phenotype, this isoform may be considered as a marker for highly aggressive disease. Our study also suggests that the 47 kDa CXCR4 could be a potential molecular target for drug development, as marked decrease in the invasion index of highly invasive cells could be achieved by blocking the 47 kDa CXCR4 isoform.

Cell lines Inden, MNB-OZ and MNB-SU were established at our institute, and NB Mass, at our collaborative institute. NB Mass was derived from a 7 month old boy with stage 1 disease, diagnosed by the mass screening system for neuroblastoma at 6 months of age. N-myc amplification was absent in the tumor. The patient was induced to complete remission of the disease with surgery and chemotherapy [41]. In agreement with the clinical features presented by the patient, the cell line was very less invasive, and did not express the 47 kDa CXCR4 isoform. Cell line Inden, was generated from a primary tumor in the adrenal gland of a 4 year 10 month old patient. The disease was classified as stage 4 and no n-myc amplification was observed. Remission was achieved with surgery and chemotherapy. This cell line showed low invasion index and did not express the 47 kDa CXCR4 isoform. MNB-SU and MNB-OZ were generated from stage 4 highly aggressive tumors refractory to treatment. These two patients were not induced to complete remission. These cell lines showed highly invasive characteristics, and expressed the 47 kDa CXCR4 isoform. This reveals a correlation between the disease aggressiveness presented in the patients, and the expression of the 47 kDa CXCR4 isoform and invasion index of the cell line. However, we did not find any correlation between N-myc amplification and the invasive potential of the cell lines (Table 1). Most of the highly invasive cell lines showed N-myc amplification, while no N-myc amplification was observed in 2 highly invasive cell lines KP-N-SI and SK-N-SH.

In this study we classified the cell lines into highly invasive cell lines and less invasive cell lines based on the number of cells migrating across the mesh. Cell lines with a higher than mean number of cells cell migrating across the mesh was classified as highly invasive. A second classification was made on the basis of the invasion index calculated for each cell lines. Both classification yielded identical results. A cell line with an invasion index greater than one demonstrated an affinity towards the MSC secretome and was positively modulated by the MSC secretome. All highly invasive cells consistently generated an invasion index greater than one irrespective of the initial cell number and the incubation time. This suggests invasion index to be a better indicator of invasiveness than the absolute number of cells migrating across the mesh. Invasion index, given by the ratio of number of cells migrating towards mRPMI and the number of cells migrating towards RPMI, may be considered as a new indicator for assaying the metastatic potential to the bone marrow in neuroblastoma cells.

In our study, a cell line SJ-N-SD showed very low fluorescence with calcein AM in the presence of mRPMI, and this interfered with cell counting. This might be due to fluorescence quenching molecules secreted by the cell line in the presence of mRPMI. This effect was observed only with SJ-N-SD.

The study revealed high heterogeneity in CXCR4 expression among the 20 neuroblastoma cell lines, while CXCR5 and CXCR6 expression did not show any significant variance among the cell lines. CXCR4 is proposed to regulate the tissue-specific metastasis in many cancers [42]. CXCR4 expression levels have been shown to be very heterogeneous in various cancer types [43, 44]. A high heterogeneity of the invasive potential and expression levels of CXCR4 was found among the cell lines, in accordance with previously published data [36, 43, 45]. In our study, the 87 kDa CXCR4 was not observed in the immunoprecipitated CXCR4 fraction. This might be due to the breakdown of the oligomers during immunoprecipitaion. Earlier studies had described the expression of the heavier isoforms to be associated with high total CXCR4 expression [36], but our results did not reveal such an association. No correlation was observed between the total CXCR4 expression and the invasive potential of the cell line either.

It has been suggested that the different isoforms of CXCR4 may be associated with different functional responses [43]. The 35 kDa isoform is the un-glycosilated native isoform, which, upon N-linked glyocsylation, becomes the 47 kDa active isoform. This has been reported in lymphoblastic leukemia cells [43].

We found a correlation between the expression of the 47kDa isoform of CXCR4 and increased invasive potential towards mRPMI in neuroblastoma. Further, the neuroblastoma cell membrane was found to predominantly express the 47 kDa CXCR4 isoform, suggesting its role in CXCR4 mediated bone marrow metastasis in neuroblastoma. The lower CXCR4-CXCL12 interaction in the less invasive group may be attributed to the absence of the 47 kDa isoform. Further, it could be inferred that the 47 kDa isoform is the active form of CXCR4 in neuroblastoma cell lines, and the presence of this isoform in the cell might explain the highly invasive phenotype.

MMP-9 secretion is an important parameter in the invasion process of metastasis and its evaluation would give key insights into invasive potential in tumor cells. Integrin α3 β1 has also been shown to be important in the metastasis of different types of cancers, by regulating the production of MMP-9 [40]. Reduced integrin α3 β1 expression, could be directly related to reduced MMP-9 secretion and lower invasiveness of neuroblastoma cells, under conditions of CXCR4 blockage. This suggests a major role of the 47 kDa CXCR4 isoform in responding to the MSC secretome stimuli, in bone marrow metastasis of neuroblastoma. To our knowledge, no observations have been reported on the regulatory role of specific CXCR4 isoforms in neuroblastoma metastasis to the bone marrow.

Earlier works demonstrated the regulatory effects of stromal derived factors on CXCR4 using mouse derived stromal cells in breast cancer. In our study, we found that human-MSC secretome up-regulated the expression of CXCR4, especially that of the 47 kDa isoform in neuroblastoma. A similar up-regulation of CXCR4 in response to CXCL12, mediated through NF-κB, has been reported in prostate cancer cells [46]. This up-regulation of 47 kDa CXCR4, can be considered as one of the initial steps in the sequential and specific metastasis process in neuroblastoma.

Treatment with MSC secretome, also increased CXCL12-CXCR4 binding, and up-regulated the expression of integrin α3 and integrin β1 and increased MMP-9 secretion. This may be brought about through the activation of downstream protein kinase B (AKT)/mitogen-activated protein kinase (MAPK) signaling pathway, leading to alteration of gene expression, actin polymerization, cell skeleton rearrangement and cell migration [47]. This might results in the increased invasive potential in neuroblastoma cells treated with mRPMI. Pre-treatment with CXCL12 alone also showed an increase in invasive potential with the highly invasive cell lines, but this increase was marginal compared to that with MSC secretome treatment. This suggests possible involvement of factors other than CXCL12 in the increased MMP-9 secretion and invasiveness associated with neuroblastoma cells in the presence of MSC secretome. In contrast, cell line IMR 32 showed a reduced invasion index on pretreatment with mRPMI and CXCL12. This might be the effect of down-regulation of CXCR4 in the presence of stromal factors, which has been reported earlier with some cell lines [28].

Post translational modifications have been suggested as the reason for the structural heterogeneity of CXCR4. Ubiquitination and oligomerization have been proposed as the major modifications contributing to this effect. Ubiquitination has been shown to have an antagonistic effect on the CXCR4 integrity [48, 49]. Our observations revealed increased ubiquitination of CXCR4 in the absence of the MSC secretome, suggesting a protective role of MSC secretome on CXCR4. These observations highlights a potential mechanism by which factors from the MSC protect G-protein coupled receptors (GPCRs), like CXCR4, from ubiquitination and proteasome mediated degradation.

In conclusion, this study suggests a modulatory role of the MSC secretome in the selective expression of the 47 kDa CXCR4 isoform, and in the bone marrow metastasis of neuroblastoma cells. Among the cell lines, we were able to trace the details of the clinical courses of four cell lines. These cell lines showed good correlation between the expression of the 47 kDa CXCR4 isoform and clinical aggressiveness, as well as *in vitro* invasiveness, suggesting that the 47 kDa CXCR4, up-regulated by MSC secretome may mediate metastasis of neuroblastoma into the bone marrow *in vivo*. Since, the 47 kDa CXCR4 isoform is expressed only in the highly invasive cell lines, it may be used as a marker for bone marrow metastasis in neuroblastoma. Furthermore the inhibition of the 47 kDa CXCR4 isoform should be considered as a potential strategy for therapeutic intervention. Exogenous ubiquitination of CXCR4, rendering the CXCR4 inactive with respect to metastasis, might also be considered as a promising target in neuroblastoma treatment.
