# Correction:Mesenchymal Stromal Cell Secretome Up-Regulates 47 kDa CXCR4 Expression, and Induce Invasiveness in Neuroblastoma Cell Lines

**DOI:** 10.1371/journal.pone.0167874

**Published:** 2016-12-01

**Authors:** Vipin Shankar, Hiroki Hori, Kentaro Kihira, Lei Qi, Hidemi Toyoda, Shotaro Iwamoto, Yoshihiro Komada

The fourth author’s name is incorrect. The correct name is: Lei Qi. The correct citation is: Shankar V, Hori H, Kihira K, Qi L, Toyoda H, Iwamoto S, et al. (2015) Mesenchymal Stromal Cell Secretome Up-Regulates 47 kDa CXCR4 Expression, and Induce Invasiveness in Neuroblastoma Cell Lines. PLoS ONE 10(3): e0120069. doi:10.1371/journal.pone.0120069
